# Treatment of recurrent hernia in peritoneal dialysis patients

**DOI:** 10.1093/jscr/rjad592

**Published:** 2023-10-25

**Authors:** Long Hao, Xiaoming Hong, Hongcun Sha, Yu Zhao

**Affiliations:** Department of General Surgery, Ningbo Yinzhou No.2 Hospital, Ningbo 315000, China; Department of General Surgery, Ningbo Yinzhou No.2 Hospital, Ningbo 315000, China; Department of General Surgery, Ningbo Yinzhou No.2 Hospital, Ningbo 315000, China; Department of Nephrology, Ningbo Yinzhou No.2 Hospital, Ningbo 315000, China

**Keywords:** recurrent hernia, peritoneal dialysis, laparoscopic

## Abstract

Peritoneal dialysis (PD) is the most commonly used treatment for patients with end-stage renal disease and has the advantages of simple operation and low treatment costs. However, long-term PD may lead to inguinal hernia formation, which needs to be repaired as early as possible. There are many studies on this kind of hernia, but there are few reports about how to treat recurrent hernia in PD patients. Therefore, we present a case of a female PD patient who suffered from a recurrent femoral hernia after primary hernioplasty. We successfully proceeded with treatment by laparoscopic transabdominal preperitoneal hernia repair. The patient was scheduled to receive temporary haemodialysis until the normal PD dose was restored. After 36 months of follow-up, we found that there was no recurrence of hernia, and the function of the PD catheter was normal.

## Introduction

Peritoneal dialysis (PD) is the most commonly used treatment for patients with end-stage renal disease and has the advantages of simple operation and low treatment costs [1]. However, long-term PD may lead to inguinal hernia formation, which needs to be repaired as early as possible. The Liechtenstein technique is the main repair method, which causes little harm to the peritoneum [2, 3]. However, femoral hernia may recur after primary hernioplasty especially in female patients [4, 5], which indicates that it is easy to ignore careful exploration of the femoral foramen at the time of open inguinal hernia operation. There are few reports about how to treat recurrent femoral hernia in PD patients. We successfully proceeded with treatment by laparoscopic transabdominal preperitoneal (TAPP) hernia repair for a female PD patient who suffered from a recurrent femoral hernia. The postoperative dialysis scheme was introduced.

## Case report

The patient was a 66-year-old woman with chronic renal insufficiency and hypertension. She had a medical history of laparoscopic ovarian cyst resection 10 years prior. She visited our department because she found a recurrent and reducible lump in her left inguinal region that had been present for 7 days but without abdominal pain. Previously, the patient developed a left inguinal hernia 1 month after starting PD. She immediately underwent mesh-plug repair under spinal anaesthesia in another hospital, and an indirect left inguinal hernia was found during that operation. The mesh type used was ULTRAPRO Plug (Johnson Inc., Robert-Koch-Strasse1, 22851 Norderstedt, Germany), which contained two meshes that were bridged together and were placed in a preperitoneal and overlay fashion similar to a Lichtenstein repair. The patient gradually transitioned from low-dose PD to normal-dose PD after the operation. However, 4 months after hernioplasty, a lump ~2.0 cm × 1.5 cm in size protruded from her left inguinal region again, and the size of the lump was able to change with her body position and abdominal dialysate volume. After hospitalization, the results of laboratory examination showed that the brain natriuretic peptide level was 4210.0 ng/L; serum creatinine, 668 μmol/L; and blood urea nitrogen, 16.1 mmol/L. No positive results were found in a bacterial culture of the abdominal dialysate. Ultrasonic examination suggested the patient had a left inguinal hernia. The patient’s 24-hour urine output was 700–1000 mL, and the total amount of dialysate per day was 4000 mL.

**Figure 1:**
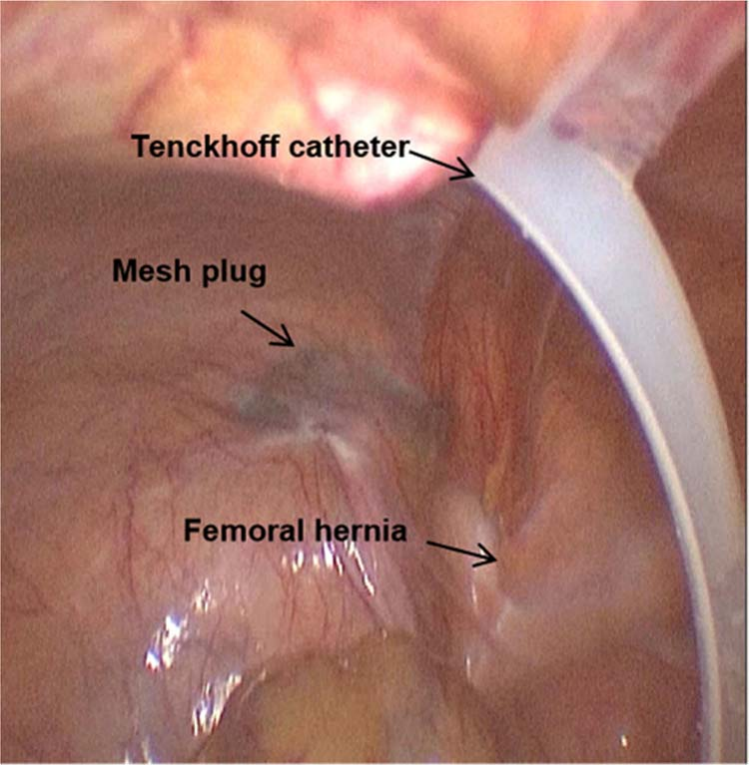
The mesh plug, Tenckhoff catheter, and femoral hernia are observed by laparoscopy.

**Figure 2:**
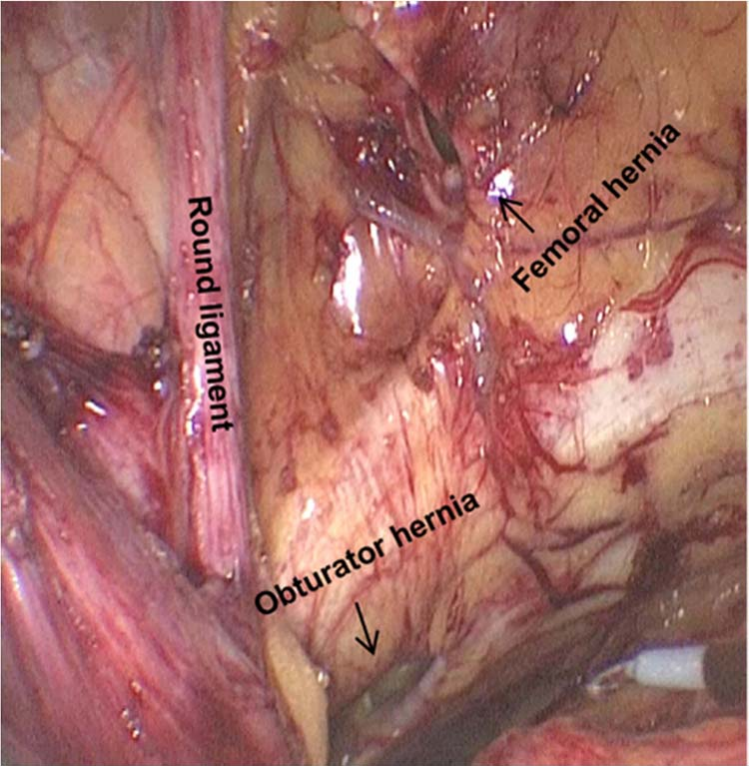
Femoral hernia and obturator hernia can be found after the medial dissection over the space of Retzius.

**Figure 3:**
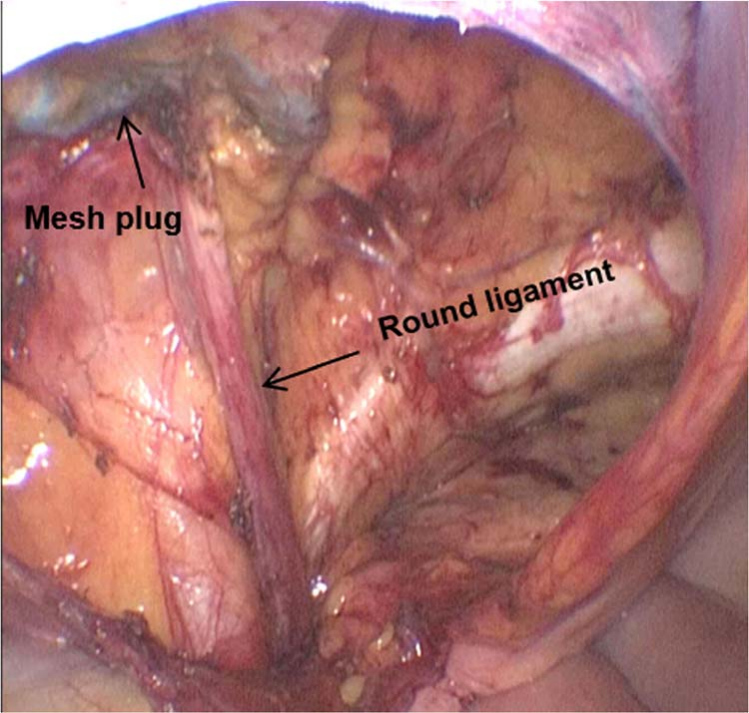
The dissected myopectineal orifice prior to mesh placement.

The patient received prophylactic antibiotic treatment during anaesthesia induction. After general anaesthesia and the successful establishment of pneumoperitoneum, we found a previously used preperitoneal mesh plug with a diameter of ~4 cm in the left inguinal region by laparoscopy (Fig. 1). We dissected the peritoneum 1 cm above the plug and carefully separated the adhesion between the peritoneum and plug. The round ligament of the uterus was preserved. After medial dissection over the space of Retzius, we found a femoral hernia and an obturator hernia during the operation (Fig. 2). When the myopectineal orifice was completely exposed (Fig. 3), a polypropylene mesh (3DMAX™ Light® Mesh 10 × 15 cm; Bard Inc., 100 Crossings Boulevard, Warwick, RI, 02886, USA) was used to overlay the entire myopectineal orifice, and the mesh was fixed on the ligamentous attachments at the pubis symphysis with 3-0 Vicryl suture. Finally, the peritoneum was tightly reconstituted by continuous suture, and the previously implanted mesh was not removed.

The patient started PD 7 days after the operation, and PD was performed in the supine position, with 500 mL fluid indwelled in the abdominal cavity for 1 hour each time. The total amount of dialysate was 2000 mL per day. Twelve days after the operation, 1000 mL of fluid was indwelled in the abdominal cavity for 2 hours each time, with a total amount of 2000 mL per day. Fourteen days later, the patient returned to a normal dose of 4000 mL per day, and 1000 mL fluid was indwelled in the abdominal cavity for 2 hours each time. Because recent irregular PD might lead to internal environment disorder and heart failure, she was scheduled to receive temporary haemodialysis until the normal PD dose was restored.

## Discussion

Considering the protection of the peritoneum, the reported PD patient did not undergo laparoscopic surgery in primary hernioplasty. And the original operation did not include high ligation of the hernia sac. The reoperation for a recurrence should be conducted via a contrary surgical approach according to the International Guidelines for Groin Hernia Management [6], so we treated the patient by laparoscopic TAPP, which can avoid the influence of scar tissue caused by anterior repair. Close suturing of the peritoneum is very important to ensure that patients can undergo PD again at an early stage, which can prevent leakage of the dialysate. It has been reported in the literature that end-stage renal disease patients can undergo early PD after open anterior mesh repair [7, 8]. For the reported patient, it takes ~1 week to receive PD again after laparoscopic TAPP, and it gradually changes from a small dose of dialysate to normal demand. PD in the supine position can reduce the pressure of peritoneal dialysate on the mesh. After 36 months of follow-up, we found that there was no recurrence of hernia, and the function of the PD catheter was normal.

In a word, we suggested that open hernia repair be used to treat inguinal hernia caused by PD, and we cannot ignore the exploration of femoral foramen at the time of the operation. Laparoscopic TAPP could be used to treat recurrent femoral hernia in PD patients.

## Data Availability

The authors declare that data supporting the findings of this study are available within the article.

## References

[1] Sun ML , Zhang Y, Wang B. et al. Randomized controlled trials for comparison of laparoscopic versus conventional open catheter placement in peritoneal dialysis patients: a meta-analysis. BMC Nephrol 2020;21:60–9. 32093633 10.1186/s12882-020-01724-w PMC7038608

[2] Horvath P , Königsrainer A, Mühlbacher T. et al. Hernia repair and simultaneous continuous ambulatory peritoneal dialysis (CAPD) catheter implantation: feasibility and outcome. Hernia 2020;24:867–72. 31773549 10.1007/s10029-019-02086-5

[3] Kou HW , Yeh CN, Tsai CY. et al. Clinical benefits of routine examination and synchronous repair of occult inguinal hernia during laparoscopic peritoneal dialysis catheter insertion: a single-center experience. Hernia 2021;25:1317–24. 33548007 10.1007/s10029-020-02364-7 PMC8514383

[4] Simons MP , Aufenacker T, Bay-Nielsen M. et al. European Hernia Society guidelines on the treatment of inguinal hernia in adult patients. Hernia 2009;13:343–403. 19636493 10.1007/s10029-009-0529-7 PMC2719730

[5] Coelho JCU , Hajar FN, Moreira GA. et al. Femoral hernia: uncommon, but associated with potentially severe complications. Arq Bras Cir Dig 2021;34:e1603. 34669892 10.1590/0102-672020210002e1603 PMC8521781

[6] The HerniaSurge Group . International guidelines for groin hernia management. Hernia 2018;22:1–165. 10.1007/s10029-017-1668-x PMC5809582 29330835

[7] Pérez-Köhler B , Bayon Y, Bellón JM. Mesh infection and hernia repair: a review. Surg Infect (Larchmt) 2016;17:124–37. 26654576 10.1089/sur.2015.078

[8] Boyer A , Bonnamy C, Lanot A. et al. How to manage abdominal hernia on peritoneal dialysis. Nephrol Thern 2020;16:164–70. 10.1016/j.nephro.2019.07.331 32001162

